# Data on *in vitro* and *in vivo* cell orientation on substrates with different topographies

**DOI:** 10.1016/j.dib.2015.09.024

**Published:** 2015-10-01

**Authors:** Andrew English, Ayesha Azeem, Kyriakos Spanoudes, Eleanor Jones, Bhawana Tripathi, Nandita Basu, Karrina McNamara, Syed A.M. Tofail, Niall Rooney, Graham Riley, Alan O׳Riordan, Graham Cross, Dietmar Hutmacher, Manus Biggs, Abhay Pandit, Dimitrios I. Zeugolis

**Affiliations:** a Regenerative, Modular & Developmental Engineering Laboratory (REMODEL), Biosciences Research Building (BRB), National University of Ireland Galway, NUI Galway, Galway, Ireland; b Network of Excellence for Functional Biomaterials (NFB), BRB, NUI Galway, Galway, Ireland; c Centre for Research in Medical Devices (CÚRAM), BRB, NUI Galway, Galway, Ireland; d School of Biological Sciences, University of East Anglia, Norwich, UK; e Centre for Research on Adaptive Nanostructures and Nanodevices (CRANN), Trinity College Dublin, Dublin, Ireland; f Materials and Surface Science Institute (MSSI), Department of Physics and Energy, University of Limerick, Limerick, Ireland; g Proxy Biomedical, Galway, Ireland; h Tyndall National Institute, Cork, Ireland; i Institute of Health & Biomedical Innovation, Queensland University of Technology, Australia

**Keywords:** Imprinting, Anisotropic substrates, Tenocytes, Subcutaneous model

## Abstract

This data article contains data related to the research article entitled “Substrate topography: A valuable *in vitro* tool, but a clinical red herring for in vivo tenogenesis” [1]. We report measurements on tenocyte viability, metabolic activity and proliferation on substrates with different topographies. We also report the effect of substrates with different topographies on host cells in a subcutaneous model.

**Specifications table**

**Table:** 

Subject area	*Biology*
More specific subject area	*Biomaterials/Tissue Engineering*
Type of data	*Figures*
How data was acquired	*in vitro assays; in vivo assays*
Data format	*Analysed data*
Experimental factors	*Substrates with various topographies*
Experimental features	*in vitro and in vivo data*
Data source location	*Galway, Ireland*
Data accessibility	*Data are supplied in this article*

**Value of the data:**

• Two-dimensional substrates, with appropriate topographical features and rigidity, may be used to maintain cell phenotype *ex vivo*.
• Two-dimensional substrates, with sub-micron to low micron features, may not be suitable for directional neotissue formation *in vivo*.
• Three-dimensional constructs may be more effective tools for directional neotissue formation *in vivo*.

## 1 Data

Herein, we assessed tenocyte viability, metabolic activity and proliferation on substrates with different topographies. The substrates were poly(lactic-co-glycolic acid) (PLGA) based with constant groove and line width of 1911.42±37.50 nm and 2101.78±35.21 nm respectively and variable groove depth of 37.48±3.4 nm, 317.29±7.05 nm and 1988.2±195.3 nm. Non-imprinted substrates were used as control. We also assessed these these substrates in a subcutaneous model.

## 2 Experimental design, materials and methods

### 2.1 Human tenocyte viability, metabolic activity and proliferation

Live/Dead® assay (BioSource International, Invitrogen, Ireland) was performed on days 1, 5 and 10 to assess cellular viability, as per manufacturer׳s protocol. Briefly, cells were washed 3 times with HBSS and exposed to the staining solution of calcein and ethidium homodimer. The cells were incubated at 37 °C for 45 min. Following staining, the cells were viewed using the BX51 Olympus fluorescence microscope and analysed using ImageJ.

Cell metabolic activity was determined using alamarBlue® assay on days 1, 5, and 10, as per manufacturer׳s protocol. Briefly, alamarBlue® dye was diluted with HBSS to make a 10% (v/v) alamarBlue® solution. Media was removed from each well and 0.5 ml alamarBlue® solution was added to each well. Cell were incubated for 3 h at 37 °C; the absorbance of the alamarBlue® was measured at wavelengths of 550 nm and 595 nm using a microplate reader (Varioskan Flash, Thermo Scientific, UK). The level of metabolic activity was calculated using the simplified method of calculating % reduction, according to the supplier’s protocol.

Cell proliferation was assessed on days 1, 5, and 10, by counting DAPI stained cell nuclei, using the BX51 Olympus fluorescence microscope.

All experiments (viability, metabolic activity and proliferation) were repeated in three independent experiments and each experiment was performed in triplicate.

### 2.2 *in vivo* study and analysis

The Animal Care Research Ethics Committee of NUI Galway approved all experimental protocols. For the subcutaneous study, female Lewis rats (200–250 g) were used, following a protocol described previously [2]. Briefly, surgery was performed on rats under general anaesthesia. Incisions were made at the back of each animal, allowing insertion of a 0.5 cm×0.5 cm structured substrate. The wound was then closed, using biodegradable sutures. Following euthanisation, the substrates were harvested at days 2 and 14 and were stained using DAPI and rhodamine conjugated phalloidin. Three animals were used per time point and at each animal all three structured substrates were implanted. Images were captured with an Olympus IX-81 inverted microscope (Olympus Corporation, Tokyo, Japan).

## 3 Results

Fig. 1, Fig. 2.

## Figures and Tables

**Fig. 1:**
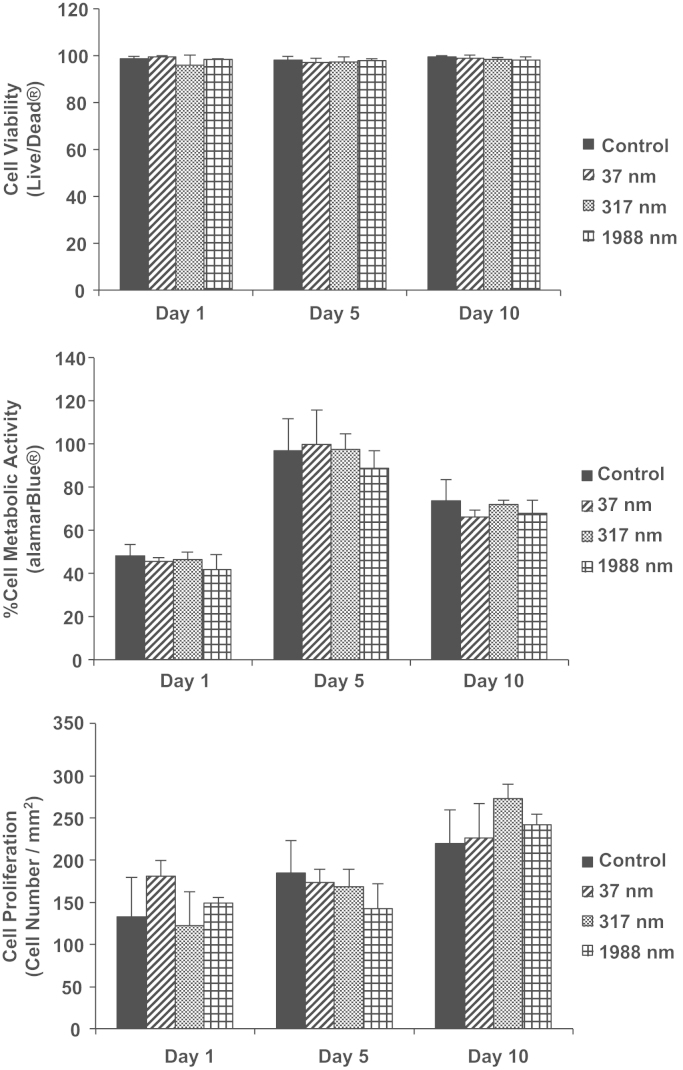
Tenocyte viability, metabolic activity and proliferation as a function of substrate topography and time in culture. No significant differences were detected.

**Fig. 2:**
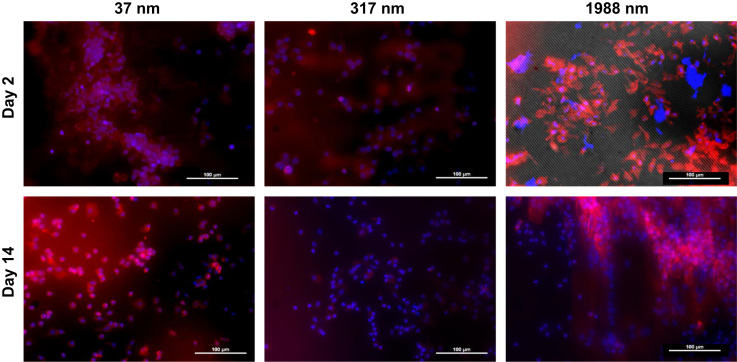
Microscopic images of host cells on substrates with different topographies. Nuclei were stained blue with DAPI and cytoskeleton was stained red with rhodamine-conjugated phalloidin. Substrate topography did not affect host cell orientation.

## References

[1] English A., Azeem A., Spanoudes K., Jones E., Tripathi B., BasuN N., McNamara K., Tofail S.A.M, Rooney N., Riley G., O׳Riordan A., Cross G., Hutmacher D., Biggs M., Pandit A., Zeugolis D.I. (2015). Substrate topography: a valuable in vitro tool, but a clinical red herring for in vivo tenogenesis. Acta Biomater..

[2] Keeney M., van den Beucken J., van der Kraan P., Jansen J., Pandit A. (2010). The ability of a collagen/calcium phosphate scaffold to act as its own vector for gene delivery and to promote bone formation via transfection with VEGF(165). Biomaterials.

